# Systematic Review of Basic Research on Alzheimer's Disease with Shen Zhi Ling Oral Liquid

**DOI:** 10.1155/2019/8216714

**Published:** 2019-04-21

**Authors:** Yahan Wang, Chunxiang Liu, Hui Wang, Yin Jiang, Pengwen Wang, Hongcai Shang

**Affiliations:** ^1^Key Laboratory of Chinese Internal Medicine of Ministry of Education and Beijing, Dongzhimen Hospital, Beijing University of Chinese Medicine, Haiyuncang Lane, Dongcheng District, Beijing 100700, China; ^2^Evidence-Based Medicine Center, Tianjin University of Chinese Medicine, Beihua South Road, Jinghai District, Tianjin 301617, China; ^3^Center for Evidence-Based and Translational Medicine, Jiangxi University of Traditional Chinese Medicine, Wanli District, Nanchang, Jiangxi 330000, China

## Abstract

**Objective:**

The present report systematically reviewed the basic research of Shen Zhi Ling oral liquid (Tiao Xin preparation) treatment on Alzheimer's disease (AD).

**Methods:**

CNKI, Wanfang, and VIP were searched, and the literature was selected according to inclusion and exclusion criteria. Data were extracted, and descriptive analysis was used.

**Results:**

Twenty-four articles were included, all of which were published as “Tiao Xin preparation.” There were seven types of AD models involved. The mechanism of action of Shen Zhi Ling oral liquid in the treatment of AD primarily included suppression of A*β* deposition and tau hyperphosphorylation, regulation of multiple neurotransmitters, improvement in energy metabolism, and promotion of the expression of autophagy-related and learning-memory-associated proteins.

**Conclusions:**

AD is a complex disease caused by multiple factors. Shen Zhi Ling oral liquid exhibited multiple and multitarget effects and great potential for treating AD. The continuous development of molecular biology and related disciplines will further elucidate the mechanism of Shen Zhi Ling oral liquid intervention in AD.

## 1. Introduction

Dementia is a chronic progressive disease that is primarily characterized by intelligence impairments. These properties enable the disease to hide and progress slowly. The prevalence of dementia increases annually. The WHO predicts 29 million dementia patients worldwide by 2020, 2/3 of whom will suffer from Alzheimer's disease (AD) [1]. AD is roughly divided into early, middle, and late stages based on cognitive severity, and the clinical proportion of each stage is approximately 4:3:3 [2]. Elderly patients in the late stage lose their ability to care for themselves, behave abnormally, and exhibit low intelligence and memory loss. The poor clinical outcomes of patients with dementia in the middle and late stages cause serious mental and economic burdens on families and society. Therefore, early intervention in patients with AD or high-risk groups is essential.

Shen Zhi Ling oral liquid (also known as Shen Gui Jian Nao oral liquid, Yang Xin Jian Nao oral liquid, Nao Rui Kang oral liquid, and Tiao Xin preparation) is the first new traditional Chinese medicine compound drug in China to be approved by the China Food and Drug Administration (CFDA) (Z20120010) for the treatment of mild-to-moderate AD. This compound consists of Codonopsis pilosula, Cinnamomi mulus, Paeonia lactiflora, Radix Glycyrrhizae Preparata, Poria, ginger, Polygala, Grassleaf Sweetflag Rhizome, Fossilia Ossia Mastodi, and common oyster shell. Shen Zhi Ling oral liquid enriches* qi*, warms* yang*, reduces phlegm, and soothes the nerves, and it is suitable for patients with* heart-qi* deficiency syndrome.

Professor Lin Shuimiao of the Shanghai Geriatric Institute of Chinese Medicine prescribed the oral liquid based on the formulas of “Kai Xin Powder” and “Ling Ren Bu Wang (unforgettable formula)” in* Handbook of Prescriptions for Emergencies*, which consists of ginseng, Poria cocos, Polygala root, and Calamus under the theory of Heart Dominating Spirit and Mind. Previous studies confirmed that Shen Zhi Ling oral liquid treats AD via the regulation of multiple central neurotransmitters, the inhibition of neuronal apoptosis and injury, and inhibition of hyperphosphorylation of tau protein [3]. The present article systematically reviews the basic research of AD treatment with Shen Zhi Ling oral liquid, analyzes the progress of research on its mechanism of action, and provides baseline data for further research and development.

## 2. Materials and Methods

### 2.1. Research Objective

This review is a basic study of Shen Zhi Ling oral liquid for AD treatment and document analyses of its mechanism of action.

### 2.2. Search Method

To ensure a full recall of the appropriated studies, “Shen Zhi Ling oral liquid,” “Shen Gui Jian Nao oral liquid,” “Yang Yin Jian Nao liquid,” “brain Ruikang oral liquid,” “Tiaoxin fang,” “Tiaoxin recipe,” or “Tiaoxin prescription” were used as search words. The following databases were searched: China Knowledge Network “China Academic Journals Network Publishing Bank” (1979~11/30/2016), Chongqing Web Information Co., Ltd. “Chinese Science and Technology Journal Database” (1989~2016), Beijing Wanfang Data Co., Ltd. “Million Party Data Resource System (1998~2016), and PubMed (until 11/30/2016).

### 2.3. Inclusion Criteria

Basic research studies of Shen Zhi Ling oral liquid use in AD rodent models were included.

### 2.4. Exclusion Criteria

Duplicate publications, clinical studies, reviews or other irrelevant publications, studies with the same drug name but different ingredients, basic research using non-AD disease models, split or effective site experimental studies, incomplete description of disease models or mechanism of action, and repeated publications of the same study were excluded.

### 2.5. Data Acquisition and Analysis

Excel was used to create a database, and retrieved articles were read one at a time. Relevant data were entered, managed, and analyzed. Descriptive analysis was used to examine the mechanism of action of Shen Zhi Ling oral liquid on AD.

## 3. Results

### 3.1. Literature Search and Filter Results

**Figure 1:**
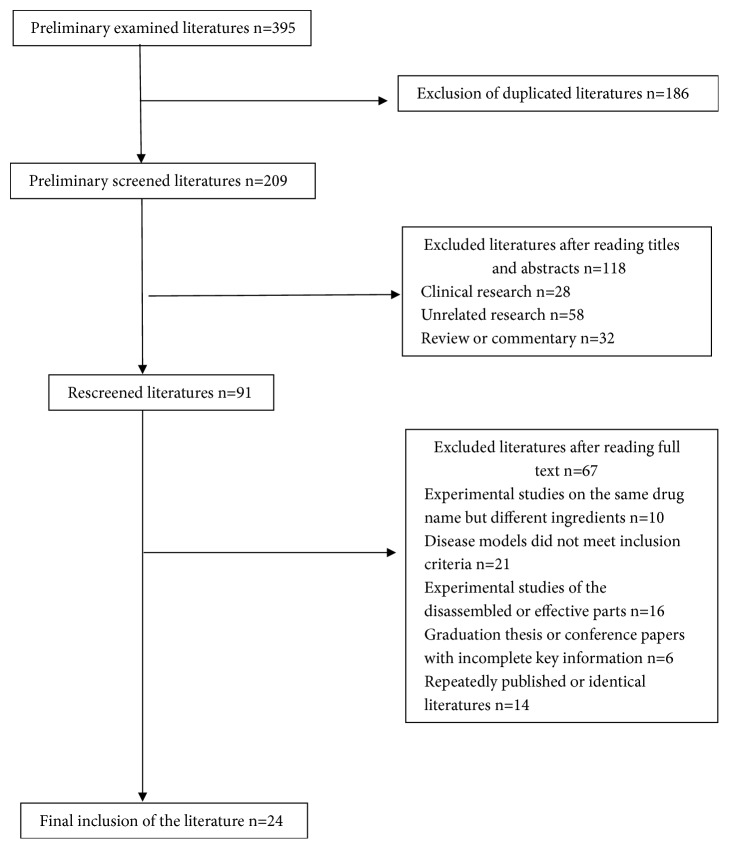
Document screening flow chart.

A total of 395 articles were initially screened out, and the remaining articles were carefully read according to the inclusion and exclusion criteria. Twenty-four articles were ultimately incorporated into the analysis. The document screening procedure is presented in Figure 1.

### 3.2. Literature Overview

The 24 articles included were published from 1998 to 2016. Most of the articles were the research of Professor Lin's team from Shanghai University of Traditional Chinese Medicine, and all of the articles were published under the name of the “Tiao Xin preparation.” These articles used eight AD models: SAMP8 mice, APP/PS1 double-transgenic mice, A*β*_1-40_ fragment left-lateral ventricle injection, A*β*_25-35_ unilateral amygdala injection, dihydroxyfumarate (DHF) and ferric chloride-adenosine diphosphate (FeCl_3_-ADP) left ventricle injection, oxidative damage due to reactive oxygen species, unilateral electrolytic lesion of the forebrain basal ganglia, and the combination of D-galactose subcutaneous injection and amanitin acid brain injection [4–28]. The details are shown in Table 1.

### 3.3. Analysis of Shen Zhi Ling Oral Liquid (Tiao Xin Preparation) on AD Rodent Models

The experiments in the literature used different rodent models, but studies on the mechanism of the drug were not affected. Tiao Xin preparation in the treatment of AD primarily included the following mechanisms of action.

#### 3.3.1. Inhibition of A*β* Deposition and Hyperphosphorylation of Tau Protein

The two major pathological features of AD are senile plaques (SPs), with A*β* serving as the main component, and neurofibrillary tangles (NFTs) being formed by hyperphosphorylated tau protein. A*β* deposition activates glial cells to secrete a variety of inflammatory cytokines and induces inflammatory responses, which further promote the expression of APP and mRNA and the deposition of A*β*, and initiates inflammation and immune cascades to induce the formation of SPs. This study found that the severity of dementia symptoms in AD patients positively correlated with the number of NFTs in brain tissue. The abnormal hyperphosphorylation of tau protein leads to the loss of the normal biological activity of catalytic microtubule assembly and stabilization of microtubule structure, and it becomes a cytotoxic molecule that promotes its own deposition as NFTs [3]. A*β* activates multiple protein kinases that overly phosphorylate tau protein to PHF-tau protein and form NFTs.

Hui Zhou et al. showed that the Tiao Xin preparation effectively inhibited the increase in the mRNA of cytokines IL-1*β* and IL-6 and APP in AD model mice [4]. Liu Xueyuan et al. confirmed that the Tiao Xin preparation reduced the expression of GFAP and APP mRNA (75l/770) in AD models, which inhibited the activation of glial cells and reduced inflammatory reactions and the large amount of amyloid-*β* protein deposition to reduce the formation of SPs [5]. Sun et al. found that the Tiao Xin preparation reduced APP gene expression in AD brains and A*β* deposition by decreasing the effects of active oxygen on NF-*κ*B [6]. Hong Daojun et al. found that the Tiao Xin preparation inhibited the expression of phosphorylated tau protein, A*β* protein, P35 protein, and cell cycle-related proteins (cyclinA and cyclinB1) in brain [7, 8]. Zhao Weikang et al. showed that the Tiao Xin preparation inhibited the expression of tau protein phosphorylation kinases (GSK-3*β* and P38 MAPK) and abnormally phosphorylated tau protein (AT-8), which reduced NFTs [9].

#### 3.3.2. Multiple Neurotransmitter Regulation

The central cholinergic system and monoamine transmitters (NE, DA, and 5-HT) are closely related to learning and memory. The central cholinergic system primarily excites the central nervous system by regulating the transition from first-level memory to second-level memory processes through two pathways, the septal-hippocampal-peripheral leaf and the cerebral cortex. Choline acetyltransferase (ChAT) is a key enzyme in the synthesis of human acetylcholine (ACh), and it is an important marker of cholinergic activity. ChAT activity is significantly reduced primarily due to cortical postsynaptic neuron degeneration, which leads to the degenerative changes of cholinergic neurons in the basal ganglia of AD patients' brains from the basal ganglia to the cortex [3]. Recent studies found that NMDARs and its subunits NR2A and NR2B were involved in the development of the central nervous system and the formation of learning and memory, and the distribution and expression of these receptors was closely related to the occurrence and development of cognitive disorders [10]. The Tiao Xin preparation increased ChAT activity in the cortex, AchE in the hippocampus, and the Rt values of M receptors and the cortical N receptors in AD models. This preparation also increased NE, DA, and 5HT in the cerebral cortex and SS and *β*-EP levels in the pituitary and plasma of AD models [11]. JinGuoqin et al. demonstrated that the Tiao Xin preparation reduced amino acid transmitter content (excitogenic amino acid transmitters (Glu, Gln, and Asp) and inhibitory amino acid transmitters (Gly, Tau, and GABA)) and the expression of NMDAR mRNA (NR1a and NR2a) for AD prevention and treatment [12].

#### 3.3.3. Energy Metabolism Improvement

Brain tissue is rich in mitochondria, and it is an important site for cellular respiration and energy metabolism. Brain tissue is also the main site for the production of oxygen free radicals. Impairment of mitochondrial respiratory function or respiratory chain complex enzyme activity creates disorder in brain energy metabolism. A large number of free radicals may damage mitochondrial structure and function, which decreases enzyme activity, endangers the energy metabolism mechanism, and affects the function of the cholinergic neurons that cause progressive learning and memory impairment in AD patients. Mitochondrial respiration III state (R3) is the rate of oxygen consumption during the fast oxygen consumption period when the substrate ADP is added. Respiration state IV (R4) is the oxygen consumption rate after ADP exhaustion in state III. The ratio of R3 and R4 is the respiratory control rate (RCR). P/O and OPR indicate the efficiency of synthesizing ATP via the mitochondrial respiratory chain releasing energy to couple ADP.

Qiu Hong et al. demonstrated that mitochondrial respiratory function (R3, R4, RCR, P/O, and OPR), complex enzyme activities (succinate dehydrogenase, NADH dehydrogenase, and CytC oxidase), and the expression of cytochrome oxidase II subunit mRNA were significantly reduced in cerebral tissue from AD model, and the Tiao Xin preparation significantly improved these indicators [13]. Sun Quan et al. showed that the activities of SOD and GSH-PX in the cortex and hippocampal subregions decreased significantly in AD models, which indicates a decreased ability of free radical scavenging in brain tissue [14]. The reason for this decrease is likely that too much reactive oxygen species (ROS) damages the oxidation of protein structures, which may reduce or inactivate enzyme activity and significantly increase malondialdehyde (MDA) content in the cerebral cortex and hippocampus. MDA is a product of the lipid peroxidation of the polyunsaturated fatty acids that are produced in ROS-attacking biofilms. The amount of MDA may reflect the degree of lipid peroxidation in the body and indirectly reflect the extent of cell damage. The abnormal changes of mitochondria in the cortex and hippocampus of rats suggest that reactive oxygen damages the ultrastructure of mitochondria. The Tiao Xin preparation increased SOD and GSH-PX enzyme activity and reduced MDA content to improve energy metabolism, protect mitochondrial structure from damage, and prevent the occurrence and development of AD. JinGuoqin et al. showed that the concentration of calcium ions in cortical and hippocampal neurons increased abnormally in AD models [15]. The overload of Ca^2+^ could further promote the lipid peroxidation and free radical formation that aggravate AD pathogenesis. The Tiao Xin preparation reduced the Ca^2+^ concentration in the cytoplasm of cortical and hippocampal neurons and improved calcium homeostasis in neurons of AD rats, which is conducive to maintaining neuronal structure and function.

#### 3.3.4. Research on Other Memory-Related Mechanism Improvement

Recent studies found that defects in autophagy may be closely related to AD occurrence and development. Model mice with removed autophagy-related genes exhibit typical neurodegenerative changes, which indicate a key role of autophagy in neurodegenerative diseases. Autophagy is the intracellular lysosome-mediated degradation of abnormally damaged proteins and organelles, and it plays an important role in maintaining homeostasis of the cell environment. Therefore, the removal of abnormally accumulated proteins in neurons via autophagy is important to maintain normal neurons. The P62 protein is a linker protein that combines with ubiquitinated proteins as a selective substrate for autophagy, and it reflects cell autophagic activity. Sanli Xing et al. showed that the Tiao Xin preparation upregulated the expression of autophagy-related protein P62 in AD models, promoted the activation of autophagy, and improved learning and memory abilities [16]. Zhenzhen Liang noticed that the Tiao Xin preparation induced the expression of learning and memory-related proteins (CaMKII and PKC) and improved the inhibition of long-term potentiation (LTP) in hippocampus [17].

## 4. Discussion

The pathological mechanism of AD is very complex, and it has not been fully elucidated to date, which hinders AD prevention and treatment research. The FDA has only approved 5 drugs for sale, and clinical studies on anti-AD drugs have been unsuccessful for a long time [29]. Basic research supports various hypotheses that the pathogenesis of AD is primarily related to the excessive deposition of amyloid protein, phosphorylation of tau protein, the mitochondrial cascade, and neuroinflammation, rather than an inevitable outcome of brain aging [30]. These studies overturned the previous understanding of the cause of AD that amyloid-*β* is good for health, and it is synthesized by the body to eliminate harmful molecules related to inflammation and immune responses, and it relieves the symptoms of AD and reverses neurodegenerative diseases, such as AD [31].

This study summarized the historical basic research on Shen Zhi Ling oral liquid treatment of AD and revealed the following main mechanisms: (1) inhibition of the excessive deposition of amyloid protein and phosphorylation of tau protein; (2) regulation of multiple neurotransmitters; (3) improving energy metabolism; and (4) improving the expression of autophagy-related proteins and learning-memory-associated proteins. An increasing number of scholars believe that the key to AD treatment lies in the early stage, especially during the symptomatic prodrome. Studies showed that the Shen Zhi Ling oral liquid regulated the expression of some AD-related genes in the hippocampus and cortex of rapidly aging mice, improved cognitive function via the removal of abnormal proteins, protected normal synaptic transmission, and regulated signal transduction and other molecular pathways [32].

There is a new Chinese medicine compound patent HTJDT-M (US patent No. 9,375,457), in which Rhizoma coptidis (RC), Cortex phellodendri (CP), and Fructus gardeniae (FG) are prepared in a dry weight ratio of 2:2:3. According to animal studies, HTJDT-M could improve the cognitive dysfunction of 3XTg-AD mice by reducing A*β* deposition, decreasing the level of detergent soluble and acid-soluble A*β* via decreasing the levels of full length amyloid-*β* precursor protein and C-terminal fragments of APP [33, 34]. In addition, it can relieve cognitive impairment caused by cerebral ischemia and disturbance of the cholinergic system [33, 35]. At present, most traditional Chinese medicines for AD are used as formulas. In addition to insomnia, diabetes, and glomerulonephritis, Liuwei Dihuang decoction and Kami-ondam-tang (KOT) could improve cognitive impairment by promoting hippocampal neurogenesis in adult rats [36, 37]. KOT can activate p-CREB, p-Akt, BDNF, and other memory-related proteins to improve the spatial memory disorder of AD [38]. Kami-shoyo-san and Danggui-Shaoyao-San treat AD by dispersing the depressed liver energy and have neuroprotective effects, which can reverse the cognitive degradation of aging mice and maintain the cerebral cortex structure [39]. Another traditional Chinese medicine formula, Fu Zheng San (FZS), consists of ginseng, radix scutellariae, calamus root, and liquorice. SAMP8 mice were used to study its effect on promoting neurogenesis. However, there is still a lack of observation on the role of this drug in the early pathological changes of AD [40]. Studies on Shen Zhi Ling oral liquid based on different kinds of mouse or rat models have demonstrated its effectiveness in the complex pathological manifestations of AD, such as A*β* deposition, nerve fiber tangles, synaptic plasticity abnormalities, myelin injury, and neuroinflammatory responses, which are more comprehensive and in depth.

AD is a complex disease caused by multiple factors, which underlies the difficulty of treatment with monomeric drugs. Chinese medicine exerts multiple effects and multiple mechanisms of action, which may produce unexpected effects for AD treatment. Treatment for AD requires considerably more research, but developments in molecular biology and its related disciplines provide an opportunity to further elucidate the pathogenesis of AD.

## Figures and Tables

**Table 1:** Basic information on the included studies.

researcher	modeling method	observation indicators	Effect of Tiao Xin preparation
Sanli Xing 2016	APP/PS1 double transgenic mice	Spatial learning and memory ability, hippocampal region p62 expression	Significantly improved the learning and memory abilities of APP/PS1 mice and increased the expression of brain hippocampus p62.

Zhenzhen Liang 2013	APP/PS1 double transgenic mice	Spatial learning and memory ability, PS peak amplitude in hippocampal CA1 region, LTP level, CaMKII, PKC protein positive expression	Improved the spatial learning and memory ability, LTP level, positive expression of CaMKII and PKC protein in the model group.

GuoqinJin 2003	A*β*_1-40_ fragment left cerebral ventricle injection	Spatial learning and memory ability, mitochondrial respiratory chain succinate dehydrogenase, NADH dehydrogenase and cytochrome oxidase activity, intracellular Ca^2+^ content in brain neurons, superoxide dismutase (SOD) activity in brain tissue, lipid peroxides product-malondialdehyde (MDA) and reactive oxygen species (ROS) content, ultrastructure of mitochondria in cerebral cortex and hippocampus	Improved the spatial learning and memory abilities, mitochondrial respiratory chain succinate dehydrogenase, NADH dehydrogenase and cytochrome oxidase activity, intracellular Ca^2+^ content of brain neurons, superoxide dismutase (SOD) in brain tissue activity, peroxidation of lipid products - Malondialdehyde (MDA) and reactive oxygen species (ROS) content, ultrastructure of mitochondria in cerebral cortex and hippocampus of AD rats in different levels.

Hong Qiu 2003	AD-like rats with oxidative injury induced by DHF and FeCl_3_-ADP injection in the left ventricle	Spatial learning and memory ability, A*β* deposition in the cortex	spatial learning and memory disorders and reduced A*β* deposition were corrected

Yi Wang 2002	A*β*_1-40_ fragment left cerebral ventricle injection	Spatial learning and memory, mitochondrial respiratory function (R3, R4, RCR, P/O, and OPR) and oxidase activity in the respiratory chain (cytochrome C oxidase, succinate dehydrogenase, NADH dehydrogenase)	Improved spatial learning and memory abilities, mitochondrial respiratory function in cortical cells and oxidase activity in the respiratory chain of model rats.

Wenxia Zhou 2002	SAMP8 mice	Gene expression of MR, tau, apoE,bcl-2,PS-2,APP,PS-1	The abnormal expression of MR, tau, and apoE in the hippocampus of 5-month-old SAMP8 mice and the abnormal expression of MR and bcl-2 in hippocampus of 12-month-old SAMP8 mice were corrected

Hui Zhou 2004	A*β*_1-40_ fragment left cerebral ventricle injection	Effect of GFAP on hippocampal and cortical astrocytes, inflammatory cytokines IL-1*β* and IL-6 mRNA levels, *β*-APP mRNA level	Decreasing the deposition of A*β* in brain tissue could induce astrocyte activation, abnormal increase of inflammatory cytokines IL-1*β*, IL-6 mRNA, and the levels of APP mRNA.

Yi Wang 2004	A*β*_1-40_ fragment left cerebral ventricle injection	Ultrastructure of mitochondria in nerve cells	Relieved the damage of ultrastructure of mitochondria in nerve cells.

Pin Chu Xu 2000	A*β*_1-40_ fragment left cerebral ventricle injection	Brain AchE activity, monoamine neurotransmitters (NE, DA, 5-HT) content	Improved the NE, DA, and 5-HT in the brain of AD model rats in different levels and no significant effect on AchE activity.

Hui Zhou 1998	spatial learning and memory impairment of AD model rats induced by *β*-amyloid protein (A*β*_1-40_) injection	Spatial learning and memory ability, cholinergic system (ChAT activity, AchE activity, M receptor) effects	Significantly improved the spatial learning and memory impairment induced by A*β* deposition. And decreasing ChAT activity and M receptor Rt values had no effect on AchE activity.

Xueyuan Liu 2005	AD-like lesion induced by A*β*_25-35_ injection in unilateral amygdala	Brain amyloid *β*-protein_1-40_, number of glial fibrillary acidic protein positive cells and mean absorbance, expression of brain APP mRNA	Decreased the amyloid expression of *β*-protein_1-40_, the expression of glial fibrillary acidic protein, and inhibited the expression of APP mRNA (751/770) in cerebral cortex and hippocampus.

Daojun Hong 2003a	AD-like lesion induced by A*β*_25-35_ injection in unilateral amygdala	Spatial learning memory, intracerebral P35 protein, and phosphorylated tau protein	The mean incubation period of water maze was shortened, and the expression of P35 and phosphorylated tau protein was decreased.

Daojun Hong 2003b	A*β*_25-35_ peptide fragment was injected into the unilateral amygdala of rats	Intracerebral AD - related proteins (phosphating tau protein, A*β* protein), intracerebral cell cycle - related proteins (cyclinA, cyclinB1)	The level of tau, A*β*, cyclinA, and cyclinB1 in the brain of model rats was decreased.

Xueyuan Liu 2004	Single side almond nucleus was injected into A*β*_25-35_ in AD rats	Spatial learning and memory ability, ChAT system (ChAT activity and M receptor), deposition of A*β*_1-40_, abnormal phosphorylation of tau protein AT8, and expression of *β*-APP mRNA	Significantly improved spatial learning and memory disorder of AD model rats, improved the ChAT activity and the M receptor Rt value, reduced APP mRNA expression and A*β*_1-40_ deposition, inhibited the abnormal phosphorylation of tau protein AT8.

Weikang Zhao 2001	Single side almond nucleus was injected into A*β*_25-35_ in AD rat models	Spatial learning and memory function of AD model rats, A*β*_1-40_, GFAP,AT-8, and PHF-1, APP770/75lm RNA, TPK-l/GSK-3*β*, P38MAPKmRNA, compared with donepezil, the regulation effect of Tiao Xin preparation was discussed	The spatial learning and memory capacity of the model rats were significantly improved, the number of GFAP positive cells, the expression of A PP770/ 751 mRNA, and the deposition of A*β*_1-40_ were decreased, tau protein phosphorylated kinase was inhibited, and tau protein abnormal phosphorylation was reduced.

Hong Qiu 2002a	AD-like rats with oxidative injury induced by DHF and FeCl_3_-ADP injection in the left ventricle	Spatial learning and memory, brain mitochondrial respiration (R3, R4, RCR, P/O, OPR), and oxidase activity (succinate dehydrogenase, NADH dehydrogenase, and CytC oxidase)	The spatial learning and memory capacity, brain mitochondrial respiration function (R3, R4, RCR, P/O, OPR, succinate dehydrogenase, NADH dehydrogenase) and CytC oxidase activity were significantly improved in the model mice.

Hong Qiu 2002b	AD-like rats with oxidative injury induced by DHF and FeCl_3_-ADP injection in the left ventricle	Mitochondrial respiration in the brain (R3, R4, RCR, P/O, OPR), composite enzyme activity (succinate dehydrogenase, NADH dehydrogenase, and CytC oxidase), cytochrome oxidase II mRNA expression	Improved mitochondrial respiratory function (R3, R4, RCR, P/O, OPR and succinate dehydrogenase, NADH dehydrogenase), the activity of cytochrome C oxidase and the expression of cytochrome oxidase II mRNA.

Guoqin Jin 2003	AD-like SD male rats with oxidative injury induced by DHF and FeCl_3_-ADP injection in the left ventricle	Brain mitochondrial respiratory chain chlorinase activity (succinate dehydrogenase, NADH dehydrogenase, and CytC oxidase), brain amino acid delivery (Glu, Gin, Asp, Gly, Tau, GABA), expression of NMDAR(NR1a and NR2a) mRNA in cerebral cortex	The content of amino acid delivery and the expression of NMDAR(NR1a and NR2a) mRNA in the brain tissue of the model rats were decreased, and the activity of mitochondrial respiratory chain cell pigment oxidase was increased.

Quan Sun 2003a	AD-like rats with oxidative injury induced by DHF and FeCl_3_-ADP injection in the left ventricle	Spatial learning and memory ability, SOD, GSH-PX activity and MDA content, and ultrastructure of cerebral cortex and hippocampal mitochondria	The abnormal changes of spatial learning and memory capacity and mitochondrial ultrastructure in the model rats were significantly improved, the activity of brain SOD and GSH-PX enzyme and the content of MDA in the model rats were significantly increased.

Quan Sun 2003b	AD-like SD male rats with oxidative injury induced by DHF and FeCl_3_-ADP injection in the left ventricle	Spatial learning and memory ability, SOD, GSH-PX activity and MDA content, and ultrastructure of cerebral cortex and hippocampal mitochondria	Improved the spatial learning and memory capacity of AD model mice, and inhibited the levels of NF-*κ*B transcription factor and APP mRNA in brain tissue.

Guoqin Jin 2001	AD-like rats with oxidative injury induced by DHF and FeCl_3_-ADP injection in the left ventricle	Spatial memory ability, brain mitochondrial respiratory chain energy metabolism enzyme (cytochrome oxidase) and succinate dehydrogenase, NADH dehydrogenase activity, type II cytochrome oxidase subunit mRNA expression level, cranial nerve yuan Ca^2+^ concentration and cerebral cortex in A*β* deposition	Significantly improved spatial memory ability of AD rats, pigment oxidase activity, and the Ca^2 +^ concentration in brain cells significantly. Lowered the number of positive cells associated with A*β* deposition in cerebral cortex and the average optical density of DAB staining.

Quan Sun 2002	Reactive oxygen AD rat models induced with oxidative damage	Spatial memory ability, activity of brain antioxidant enzyme (MDA), NF- acetyb transcription factor level, Glu, Gin and Asp content, mitochondrial ultrastructure	The activity of antioxidant enzymes in the brain was improved. The MDA generation, the active oxygen activation of the NF-kB, the deposition of A*β*, and ROS-induced excitatory amino acids toxicity were reduced. APP gene expression was lower. Calcium imbalance was prevented. Mitochondria structure was protected. Energy metabolism was improved.

Weikang Zhao 1998	The “dementia” rat models caused by unilateral electrolysis in the basal ganglia of forebrain in mice	Memory function, the cholinergic system (ChAT, AchE, M and N receptors), single amine neurotransmitter (NE, DA and 5HT) and some neuropeptides (SS and *β*- EP), and memory ability of model rats and natural aging mice	Significantly improved memory function of rat models. Improved ChAT activity in cortex and AchE activity in hippocampus and Rt value of M receptors and N receptors in cortex. Significantly increased NE, DA, and 5HT content in cerebral cortex and SS and *β*-EP in pituitary and plasma. Significantly improved memory function and learning ability of aging mice.

Yaming Li 2000a	The AD rat models were induced by the combination of D-galactose hypodermic injection and goosenic acid brain tissue directional injection	Ability of learning and memory, level of *β*-AP	Improved the learning and memory impairment of rats and decreased *β*-AP in intracerebral tissue.

Yaming Li 2000b	The AD rat models were induced by the combination of D-galactose hypodermic injection and goosenic acid brain tissue directional injection	Ability of learning and memory, level of acidified tau protein	Improved learning and memory impairment in rats. Decreased phosphorylation of tau protein levels in brain tissue.

Yaming Li 2000c	The AD rat models were induced by the combination of D-galactose hypodermic injection and goosenic acid brain tissue directional injection	Ability of learning and memory, cholinergic system (ChAT, AchE, M receptors)	Rt value of M receptor, activity of AchE and ChAT in cortex were upregulated. The learning and memory ability of mice was improved.

Some publications were different reports from the same author on the same study in the same year, which are distinguished by a, b, and c.

## Abbreviations

A *β*: Amyloid *β*
GFAP: Fibrillary acid protein
Il-1*β*: Interleukin -1*β*
Il-6: Interleukin-6
*β*-APP mRNA: Amyloid protein precursor protein gene
AchE: Acetylcholinesterase
NE: Norepinephrine
DA: Dopamine
5-ht: 5-Hydroxytryptamine
ChAT: Acetylcholine transferase
Glu: Glutamate
Gin: Glutamine
Asp: Aspartic acid
Gly: Glycine
Tau: Taurine
GABA: Constant-aminobutyric acid
SOD: Superoxide dismutase
GSH - PX: Glutathione peroxidase
MDA: Malondialdehyde
SS: Somatostatin
*β*-EP: Abolitionists - endorphins
NMDAR: n-methyl d-aspartic acid receptor
MR: Mineralocorticoid receptor
Apo E: Apolipoprotein E
Bcl-2: B-cell lymphoma-2
PS1: Presenilin-1
PS2: Presenilin-2
p-CREB: Phosphorylated cyclase response element binding protein
p-Akt: Protein kinase B
BDNF: Brain derived neurotrophic factor.
